# Plant odorants interfere with detection of sex pheromone signals by male *Heliothis virescens*

**DOI:** 10.3389/fncel.2012.00042

**Published:** 2012-10-08

**Authors:** Pablo Pregitzer, Marco Schubert, Heinz Breer, Bill S. Hansson, Silke Sachse, Jürgen Krieger

**Affiliations:** ^1^Institute of Physiology, University of HohenheimStuttgart, Germany; ^2^Department of Evolutionary Neuroethology, Max Planck Institute for Chemical EcologyJena, Germany; ^3^Department of Biology, Chemistry and Pharmacy, Institute of Biology/Neurobiology, Free University BerlinBerlin, Germany

**Keywords:** pheromone detection, antennal lobe, pheromone receptor, pheromone binding protein, olfaction

## Abstract

In many insects, mate finding relies on female-released sex pheromones, which have to be deciphered by the male olfactory system within an odorous background of plant volatiles present in the environment of a calling female. With respect to pheromone-mediated mate localization, plant odorants may be neutral, favorable, or disturbing. Here we examined the impact of plant odorants on detection and coding of the major sex pheromone component, (Z)-11-hexadecenal (Z11-16:Ald) in the noctuid moth *Heliothis virescens*. By *in vivo* imaging the activity in the male antennal lobe (AL), we monitored the interference at the level of olfactory sensory neurons (OSN) to illuminate mixture interactions. The results show that stimulating the male antenna with Z11-16:Ald and distinct plant-related odorants simultaneously suppressed pheromone-evoked activity in the region of the macroglomerular complex (MGC), where Z11-16:Ald-specific OSNs terminate. Based on our previous findings that antennal detection of Z11-16:Ald involves an interplay of the pheromone binding protein (PBP) HvirPBP2 and the pheromone receptor (PR) HR13, we asked if the plant odorants may interfere with any of the elements involved in pheromone detection. Using a competitive fluorescence binding assay, we found that the plant odorants neither bind to HvirPBP2 nor affect the binding of Z11-16:Ald to the protein. However, imaging experiments analyzing a cell line that expressed the receptor HR13 revealed that plant odorants significantly inhibited the Z11-16:Ald-evoked calcium responses. Together the results indicate that plant odorants can interfere with the signaling process of the major sex pheromone component at the receptor level. Consequently, it can be assumed that plant odorants in the environment may reduce the firing activity of pheromone-specific OSNs in *H. virescens* and thus affect mate localization.

## Introduction

The ability of many insect species to use plant volatiles and pheromones to locate food, sexual partners, and appropriate egg-laying places is crucial for survival and reproduction (Zwiebel and Takken, 2004; Vosshall, 2008; Carey and Carlson, 2011; Hansson and Stensmyr, 2011). The remarkable pheromone detection system of male moths (Schneider, 1992; Hansson, 1995) allows them to recognize female-released sex pheromone blends from long distances (David et al., 1983; Vickers and Baker, 1997); in addition, it triggers and controls upwind flight behavior and guides the sexual partner to the calling female (Vickers et al., 1991; Vickers, 2006; Carde and Willis, 2008).

Components of female sex pheromone blends are detected by specialized sensilla on the male antenna (Almaas and Mustaparta, 1991; Baker et al., 2004). These porous, hair-like structures house the dendrites of pheromone-responsive olfactory sensory neurons (Ph-OSNs) bathed in sensillum lymph containing a high concentration of pheromone binding proteins (PBP) (Vogt and Riddiford, 1981; Steinbrecht and Gnatzy, 1984; Zhang et al., 2001). Specific PBPs take distinct pheromone molecules from the air and transfer them through the lymph toward specific pheromone receptors (PRs) in the dendritic membrane of Ph-OSNs (Leal, 2003; Vogt, 2003; Grosse-Wilde et al., 2006, 2007; Forstner et al., 2009). The Ph-OSNs for different pheromone components are endowed with specific PRs (Krieger et al., 2004; Nakagawa et al., 2005; Grosse-Wilde et al., 2010; Wanner et al., 2010) and converge their axons into separate compartments of the macroglomerular complex (MGC), the male-specific pheromone-processing center within the antennal lobe (AL) (Hansson et al., 1992; Berg et al., 1998; Hansson and Anton, 2000). In contrast, signals from general odorants, e.g., plant volatiles, are detected by general OSNs and transferred to sexually isomorphic ordinary glomeruli in the AL (Galizia et al., 2000; Hansson et al., 2003).

When they are released the female-produced sex pheromones are embedded within a background of general odorants, mainly plant volatiles. The air concentration of odorants depends on various environmental parameters: the abundance of vegetation, time of day, and weather (Kesselmeier and Staudt, 1997; Müller et al., 2002). Therefore, a male's sex pheromone detecting system is exposed simultaneously to mixtures of pheromone components and general odorants at varying ratios. The highest concentrations of both pheromone and plant odorants are likely to be present near to the calling female, which is often situated on a host plant. Although many studies of insect olfaction have addressed antennal detection and central coding of single compounds, as well as of mixtures of plant odorants or of pheromone blends, (e.g., Galizia and Menzel, 2000; Galizia et al., 2000; Hallem and Carlson, 2006; Lei and Vickers, 2008; Wang et al., 2010; Kuebler et al., 2012), few studies have examined pheromone/plant odorant mixtures. Recent electrophysiological studies on the pheromone sensilla of *Heliothis virescens* (Hillier and Vickers, 2011), *Spodoptera littoralis* (Party et al., 2009) and *Agrotis ipsilon* (Deisig et al., 2012) have found that the firing activity of Ph-OSNs to specific pheromone components was suppressed when plant odorants were co-applied. In contrast, an earlier study on *Helicoverpa zea* indicated enhancement of the pheromone-evoked spike activity of Ph-OSNs in the presence of plant odorants (Ochieng et al., 2002). Stimulation of the antenna with the plant odorant heptanal was found to reduce the pheromone response in the MGC of *Agrotis ipsilon* on both the input (Ph-OSNs) and output side (projection neurons, PNs) (Chaffiol et al., 2012; Deisig et al., 2012); conversely, in the silk moth *Bombyx mori*, sex pheromone responses in PNs of the MGC were enhanced in the presence of the host plant odor Z3-hexenol (Namiki et al., 2008).

Current data indicate that the interference of plant odorants with pheromone responses may appear already at the level of antennal sensilla—suppressing or enhancing Ph-OSN firing activity which is conveyed to the AL. The molecular targets at which plant odorants may interfere with pheromone-induced activities of OSNs are unknown, but key elements involved in pheromone detection, such as PBPs in the sensillum lymph or PRs in the dendrites of Ph-ORNs, are considered as candidates (Party et al., 2009; Deisig et al., 2012). Our previous studies have indicated that an interplay of the PBP HvirPBP2 and the PR HR13 is important for eliciting cellular responses by the major sex pheromone component Z11-16:Ald (Grosse-Wilde et al., 2007). In the present study we set out to explore if plant volatiles may interact with any of these key elements. In order to identify plant odorants, which may affect pheromone detection in *H. virescens* we performed *in vivo* imaging experiments monitoring pheromone-evoked activity in the so-called cumulus region of the MGC, where Z11-16:Ald-specific OSNs terminate (Galizia et al., 2000). Using a fluorescence-based competitive binding assay we examined how identified plant odorants and pheromone/plant odorant mixtures bind to HvirPBP2. Furthermore, a cell line expressing the PR HR13 was employed in fura-2-based calcium imaging studies to test whether plant odorants interfere with Z11-16:Ald detection at the level of the PR.

## Materials and methods

### Animals

*H. virescens* pupae were kindly provided by Bayer CropScience, Frankfurt, Germany. Pupae were sexed and allowed to develop at room temperature. After emergence, moths were fed on 10% sucrose solution.

### Pheromone and plant odorants

(Z)-11-hexadecenal (Z11-16:Ald) was purchased from Fluka or Bedoukian. Plant odorants (β-caryophyllene, geraniol, Z3-hexenol, isoamyl acetate, linalool, linalyl acetate) were purchased from Fluka, Sigma, and Merck at the highest purity available. β-caryophyllene, geraniol, Z3-hexenol, and linalool were selected because of their physiological and ecological relevance to heliothine moths. For these chemicals previous studies on male and female *H. virescens* have identified responsive OSNs on the antenna, processing glomeruli in the AL or effects on behavior (De Moraes et al., 2001; Skiri et al., 2004; Hillier et al., 2005; Rostelien et al., 2005; Hillier and Vickers, 2007). Isoamyl acetate was chosen as a typical fruit odor. Linalyl acetate was selected because it is chemically related to linalool and emitted as a principle component from many flowers and spice plants. For optical imaging experiments, plant odorants were diluted in mineral oil (Sigma-Aldrich) to a concentration of 1:10 (v/v) which equates to 85–90 μg/μl. The pheromone component Z11-16:Ald was diluted to a final concentration of 1 μg/μl.

### Optical imaging of the antennal lobe

Moths were 1- to 5-day-old male *H. virescens*. Animals were gently pushed into a 1000 μl pipette whose tip had been cut open and then fixed with dental wax. After the scales were removed, the labial palps and proboscis were fixed to reduce movement artifacts. A window was cut into the head cuticle between the compound eyes. Glands and trachea were carefully removed to get access to the brain. A fluorescent calcium indicator (Calcium Green-1 or 2 AM, Invitrogen) was dissolved in Ringer solution (150 mM NaCl, 3 mM KCl, 3 mM CaCl_2_, 25 mM sucrose, 10 mM TES buffer, pH 6.9) with 6% Pluronic F-127 (Invitrogen) to a concentration of 30 μmol. The brain was incubated with ~20 μl of this solution at 4°C. After incubation for 60 min, the brain was rinsed several times with Ringer solution.

Imaging experiments were performed using a Till Photonics imaging setup (TILL imago, Till Photonics GmbH) with a CCD-camera (PCO imaging, Sensicam) and a fluorescence microscope (Olympus, BX51WI) equipped with a 20× water immersion objective (NA 0.95, XLUM Plan FI, Japan). Calcium Green™ was excited at 475 nm (500 nm SP, xenon arc lamp, Polychrome V, Till Photonics), and fluorescence was detected at 490/515 nm (DCLP/LP). The whole setup was placed on a dumping table. Fourfold binning on the CCD-camera chip gave a resolution of 1.25 μm/pixel with an image size of 344 × 260 pixels.

Six μl of plant odorants (i.e., 510–540 μg) or 10 μl of pheromone component (i.e., 10 μg) was pipetted on a filter paper (12 mm diameter), which was inserted into a glass pipette; these were renewed every day. A stimulus controller (Syntech, Stimulus Controller CS-55) was used to apply the odor in a continuous airstream, whose flow of 0.6 l/min was monitored by a flow meter (Cool Parmer). An acrylic glass tube guided the airflow to the moth's antenna. For mixture application, plant odorant and pheromone component were applied in two separate pipettes which were inserted into the continuous airstream (stimulus flow: 0.4 l/min). In case of single odor application, the second pipette was empty. Each recording had a continuance of 10 s with an acquisition rate of 4 Hz. Odors were applied after 2 s for 2 s. Single moths were imaged for up to 1 h, with interstimulus time intervals (ISI) of 1–3 min. The sequence of stimulations was randomized from insect to insect and repeated in a few cases to test for reproducibility of the odor-evoked activity patterns.

The imaging data were processed as previously described (Bisch-Knaden et al., 2012) using custom-written software in IDL (ITT Visual Information Solutions). To quantify odor-evoked calcium signals, we identified the cumulus because of its clear response to Z11-16:Ald and its proximity to the antennal nerve entrance. In each animal, the responses were normalized to the maximal response over all odorants. We defined the average of frames 10–18 (i.e., 0.5 s after stimulus onset until 0.5 s after stimulus offset) as the odor-evoked signal intensity.

### Expression and purification of *H. virescens* PBP2

The bacterial expression of *H. virescens* PBP2 (HvirPBP2) (Krieger et al., 1993) and purification of the protein from a periplasmic fraction of *E. coli* BL21 (DE3) was performed as described previously (Campanacci et al., 2001; Grosse-Wilde et al., 2007). Recombinant HvirPBP2 was delipidated to remove possible hydrophobic ligands, which may co-purify with PBP expressed in bacteria (Oldham et al., 2001), and finally dissolved in Ringer solution (138 mM NaCl, 5 mM KCl, 2 mM CaCl_2_, 1.5 mM MgCl_2_, 10 mM Hepes, 10 mM glucose, pH 7.3). The protein concentration was determined using a spectrometer at 280 nm applying the absorption co-efficient determined by the ProtParam program (ExPASy molecular biology server: http://www.expasy.org). Finally, the protein solution was aliquoted and stored at −70°C until use. Once unfrozen, the HvirPBP2 solution was kept at 8°C.

### Competitive fluorescence binding assay with HvirPBP2

To evaluate the binding of plant odorants to HvirPBP2 and an interference of plant odorants with pheromone binding, a competitive fluorescence binding assay that had previously been used to characterize ligand binding to various PBPs and odorant binding proteins (OBPs) of insects, including moth, flies, locust, and mosquitoes was applied (Campanacci et al., 2001; Ban et al., 2003; Zhou et al., 2004; Qiao et al., 2010).

Fluorescence emission spectra (360–600 nm) after excitation at 337 nm were recorded on a PerkinElmer LS 50B spectrofluorimeter using a quartz cuvette with a 1 cm light path fluorimeter in a right angle configuration and emission slit width of 5 nm were used. The binding of 1-N-phenylnapthylamine (1-NPN) to HvirPBP2 was determined by titrating 2 μM protein with increasing concentrations of the chromophore dissolved in dimethyl sulfoxide (DMSO).

For competitive binding experiments, HvirPBP2 (2 μM) in Ringer solution was loaded with 2 μM 1-NPN. The change in 1-NPN fluorescence was monitored after increasing amounts of Z11-16:Ald, plant odorants, or combinations of both from stock solutions (10 mM each; freshly prepared in methanol) were added to a final concentration of 10 μM. In control experiments, we observed no significant effects of the solvents in use (methanol for the pheromone component and plant odorants; DMSO for 1-NPN) on 1-NPN binding to HvirPBP2. To evaluate how different compounds and pheromone/plant odorant mixtures bound to HvirPBP2, the maximum 1-NPN fluorescence at a given concentration was determined and related to the maximum 1-NPN fluorescence in the absence of competitor (= 100%). For data analysis and graphic plotting, the program GraphPad Prism version 4.0 (GraphPad Software, San Diego, CA, USA) was used. The *K*_diss_ for Z11-16:Ald binding to HvirPBP2 was calculated according to *K*_diss_ = [IC50]/(1 + [1-NPN]/K_1−NPN_) with [1−NPN] = 1−NPN concentration and K_1−NPN_ = 1−NPN dissociation constant for PBP/1−NPN.

### Calcium imaging of HR13-expressing cells

To analyze the effect of plant-derived odorants on Z11-16:Ald detection by the PR HR13 (Krieger et al., 2004), we used a stable receptor-expressing cell line. The generation and functionality of HR13/Flp-In T-REx293/Gα15 cells have been described previously (Grosse-Wilde et al., 2007). HR13/Flp-In T-REx293/Gα15 cells were cultured using DMEM media (Invitrogen) supplemented with 10% fetal calf serum and either 100 mg/L hygromycin, 10 mg/L blasticidin, or 200 mg/L geneticin in regular alternation.

Calcium imaging experiments were performed as described previously (Grosse-Wilde et al., 2006, 2007; Forstner et al., 2009). Briefly, 48 h before imaging, 0.7 × 10^5^ cells were plated onto poly-L-lysine coated glass coverslips (Ø15 mm, Hecht, Sondheim, Germany), harbored in a 24 well plate. After 24 h, receptor expression was induced by adding 5 mg/ml tetracycline. Twenty-four hours later, cells were washed with warmed Ringer solution (138 mM NaCl, 5 mM KCl, 2 mM CaCl_2_, 1.5 mM MgCl_2_, 10 mM Hepes, 10 mM Glucose, pH 7.3) and incubated with 4 μmol/L fura-2 AM (Invitrogen) in Ringer solution at 37°C for 30 min. A flow chamber was used to place a coverslip with cells loaded with fura-2 onto the stage of an inverted microscope (Olympus IX70) equipped for epifluorescence. Cells were permanently rinsed with Ringer solution (warmed to 37°C) at a flow rate of 1 ml/10 s. Control and test solutions (400 μl each) were applied at the same flow rate using a three-way valve system with connected syringes.

In a single experiment, cells were first rinsed for at least 5 min with Ringer solution, after which a control stimulus was applied (Ringer solution with 0.1% DMSO and 0.1% *n*-Hexane). This procedure allowed us to monitor responses to DMSO or *n*-hexane and to eliminate spontaneously active cells from later data analysis. After rinsing cells for another 5 min with Ringer solution, test odorants were applied. Z11-16:Ald and plant odorants were diluted from stock solutions in *n*-hexane using Ringer solution with 0.1% DMSO. All dilutions were prepared freshly before imaging started. Following the application of test substances, the viability of the cell was tested by applying 10 mM ATP in Ringer solution directly to the cell chamber. To monitor changes in the intracellular Ca^2+^ concentration in individual cells, light emission at 510 nm was measured over time following excitation at 340 nm and 380 nm. Data analysis and acquisition were performed with the Metafluor imaging system and Metafluor 4 software (Visitron Systems, Puchheim, Germany). Changes in fluorescence intensity at 340 nm/380 nm excitation were used as an index of increasing calcium concentrations. Ratios of fluorescence intensity for at least 30 cells per experiment were determined before (*F*_0_) and after stimulation (*F*; peak of response). *F*/*F*_0_ values of individual cells were determined and averaged in a single experiment.

## Results

### Plant odorants affect pheromone-induced responses in the AL

**Figure 1:**
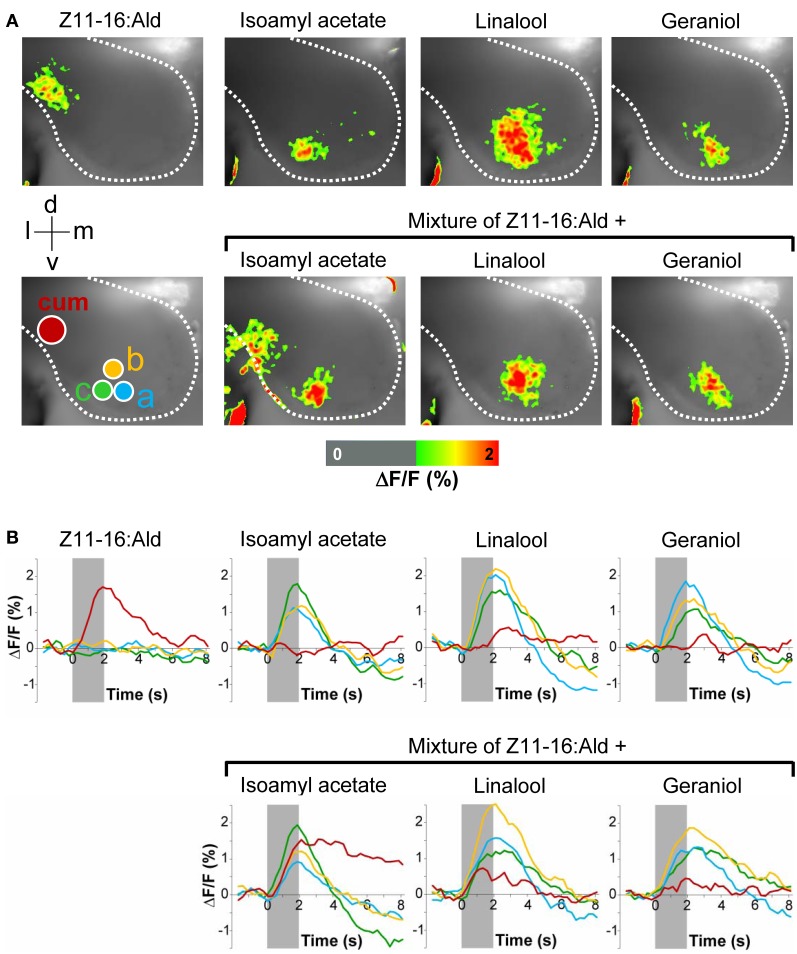
**Effect of plant odorants on pheromone-induced calcium signals in the moth antennal lobe.** The antenna of a *H. virescens* male was stimulated separately with single compounds or simultaneously with Z11-16:Ald (10 μg) and plant odorants [1:10 (v/v) diluted in mineral oil]. Activity patterns in the antennal lobe were monitored using calcium imaging. **(A)** Representative false-color coded spatial response patterns. The positions of the cumulus (cum) region in the macroglomerular complex and of three ordinary glomeruli (a–c) are indicated by colored circles. All images are scaled to the overall maximum of all measurements. Images represent Δ*F*/*F* (in % change from background) superimposed onto the raw fluorescence images according to the scale below of one representative male moth. The directions medial (m), lateral (l), dorsal (d), and ventral (v) are indicated. **(B)** Time courses of glomerular calcium responses shown as Δ*F*/*F* (in %) of the cumulus (red line) and three ordinary glomeruli (yellow, green, and blue lines) as marked with circles in **(A)**. The odor stimulation is indicated by the gray bar.

In order to analyze the interference between volatiles of host plants that are present in the environment of calling females and the major sex pheromone component Z11-16:Ald, we performed functional *in vivo* calcium imaging of the AL of male *H. virescens*. We compared odor-evoked calcium activity patterns after separately or simultaneously stimulating the antenna with Z11-16:Ald and different plant odorants. Stimulation of the antenna with Z11-16:Ald alone revealed clear calcium signals in the cumulus of the MGC, the place where Z11-16:Ald-reactive OSNs terminate (Figure 1A). In contrast, none of the plant odorants tested clearly activated the cumulus. Instead, the various plant odorants generated calcium signals in distinct yet partly overlapping sets of ordinary glomeruli (Figure 1A, upper row). This observation was substantiated in time course measurements (Figure 1B, upper row). Simultaneously stimulating the antenna with mixtures of the pheromone component and a plant odorant revealed different spatio-temporal activity patterns in the AL (Figure 1A, lower row). The combination of Z11-16:Ald and isoamyl acetate elicited calcium signals that were almost the sum of the responses obtained by stimulation with the single compounds. In contrast, application of Z11-16:Ald in combination with linalool or geraniol led to a significantly reduced pheromone-induced activity in the cumulus region, whereas the calcium response in the ordinary glomeruli appeared almost unaltered. These results were supported by the time courses of the measurements (Figure 1B, lower row).

**Figure 2:**
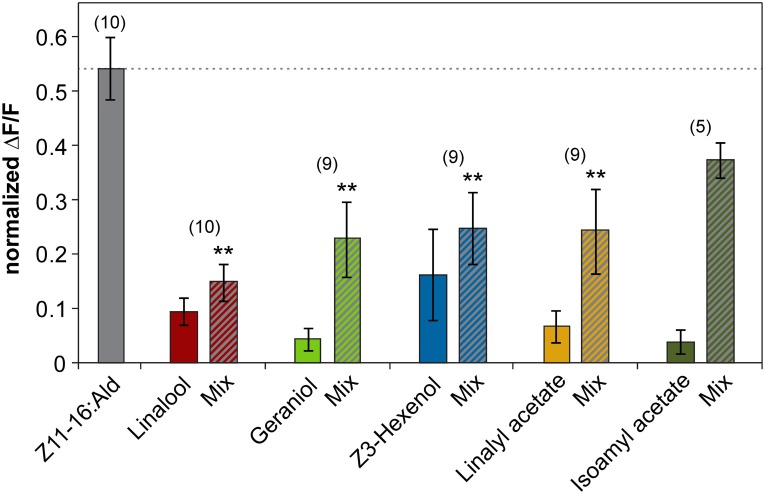
**Plant odorants inhibit the pheromone-induced activity in the cumulus region of the antennal lobe.** Relative fluorescence changes in the cumulus region of the antennal lobe upon stimulation with Z11-16:Ald (10 μg, gray bar), or with the plant odorant indicated [1:10 (v/v) diluted in mineral oil, colored bars] or with a mix of both (striped bars). Data represent the mean response including the standard error of mean (SEM) based on 5–10 *H. virescens* males. Δ*F*/*F* [%] values have been normalized for each individual over all odors and glomeruli by setting the maximum response to 1. All plant odorants except isoamyl acetate significantly reduce the pheromone-induced response in the cumulus (^**^p < 0.01; ANOVA followed by Dunnett Multiple Comparisons Test).

The inhibitory effect of linalool and geraniol onto the pheromone-induced response in the cumulus was reproducible between different individuals (Figure 2). In addition, we observed a clear inhibitory effect for the two odorants Z3-hexenol and linalyl acetate. For isoamyl acetate, a slight inhibitory effect was observed in a few animals, but did not prove to be statistically significant (Figure 2). Thus, our experiments demonstrated that several, but not all plant odorants clearly inhibit the induced activity pattern in the first processing center for pheromone signals.

### Interference of plant odorants with molecular elements of pheromone signaling

The observation that plant odorants reduce pheromone-induced spiking activity of Ph-OSNs (Party et al., 2009; Hillier and Vickers, 2011; Deisig et al., 2012) suggests that the inhibitory effects of plant odorants on the Z11-16:Ald-evoked activity we monitored in the MGC (Figures 1 and 2) may result from an interference of plant odorants with molecular elements of pheromone detection in the antenna. It has recently been shown that in *H. virescens* the Z11-16:Ald detection involves the PBP HvirPBP2 and the PR HR13 (Grosse-Wilde et al., 2007). Therefore, we asked if plant-related odorants may affect these two components of the pheromone recognition system.

### Plant odorants do not bind to HvirPBP2

**Figure 3:**
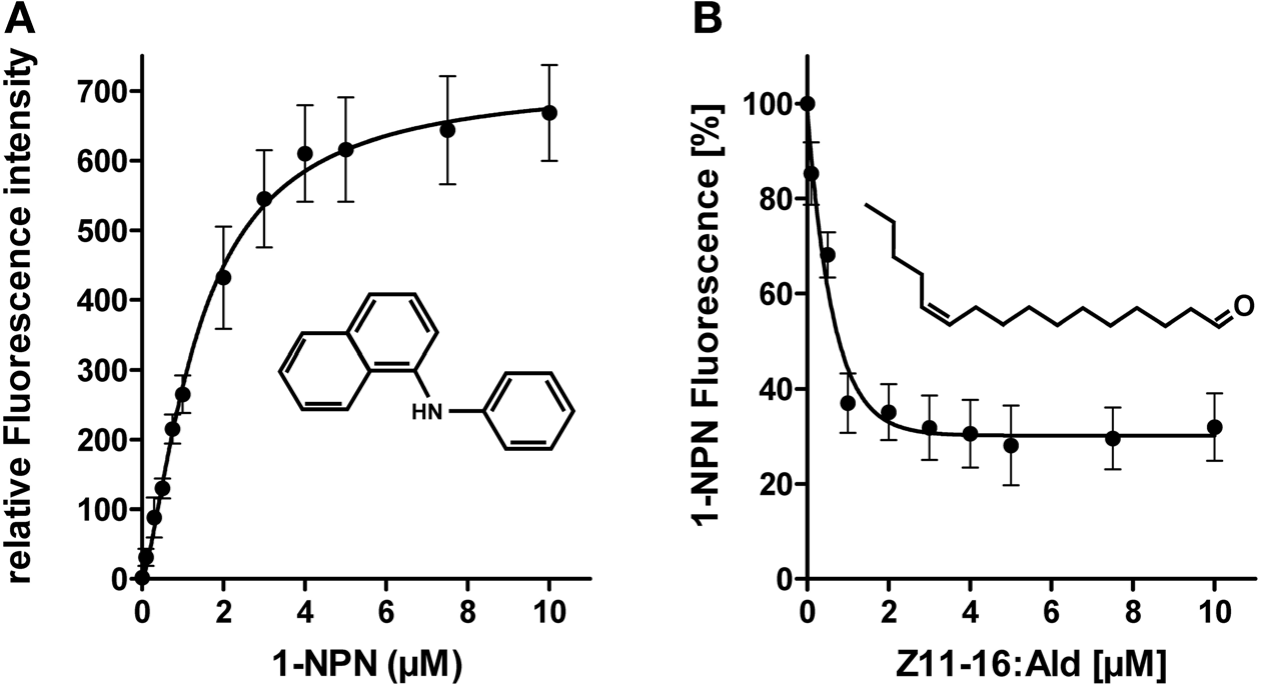
**1-NPN binds to HvirPBP2 and is displaced by Z11-16:Ald. (A)** Relative fluorescence intensity as a function of 1-NPN concentration. HvirPBP2 in Ringer solution (2 μM) was titrated with increasing amounts of 1-NPN to a final concentration of 10 μM. **(B)** Competitive fluorescence binding assay on HvirPBP2 (2 μM in Ringer solution) using 1-NPN (2 μM). Maximum emission of 1-NPN fluorescence was monitored after increasing concentrations of Z11-16:Ald (0–10 μM) were added. Fluorescence intensities at different pheromone component concentrations are shown as percentages of the maximum 1-NPN fluorescence in the absence of the pheromone component. Data represent the mean of three independent measurements. Standard deviations are indicated by error bars.

First, we have analyzed if plant odorants may be able to occupy the binding pocket of HvirPBP2 and thereby prevent binding of Z11-16:Ald. To estimate the binding of odorants to HvirPBP2, we conducted fluorescence displacement assays employing 1-NPN as fluorescence reporter. When excited at 337 nm, 1-NPN in aqueous buffer emits fluorescence only weakly. However, in a hydrophobic environment, such as the hydrophobic binding pocket of PBPs (Sandler et al., 2000), the fluorescence intensity increases and the emission maximum blue-shifts. Accordingly, the titration of 1-NPN to HvirPBP2 in Ringer solution resulted in a large increase in fluorescence intensity (Figure 3A) and a shift of the emission maximum from 465 nm to 402 nm (not shown). The concentration-dependent binding of 1-NPN can be described by a hyperbolic curve (Figure 3A), which is consistent with a one-site binding model and a calculated *K*_diss_ value of 1.4 μM.

To test the functionality of the assay system and the integrity of the purified HvirPBP2, we monitored the ability of Z11-16:Ald to displace 1-NPN (Figure 3B). We found that upon titration of the pheromone component, the 1-NPN fluorescence was reduced in a concentration-dependent manner, indicating the pheromone component had bound to the hydrophobic binding pocket of HvirPBP2. Half-maximum 1-NPN displacement was obtained at a pheromone component concentration of 0.8 μM. Calculation of the relative dissociation constant revealed a *K*_diss_ of 0.33 μM. A binding affinity for pheromones in the micromolar range was also found for the PBPs of other insects (Plettner et al., 2000; Campanacci et al., 2001). Thus, the competitive 1-NPN displacement assay demonstrated that Z11-16:Ald binds to HvirPBP2; this finding confirms and extends previous results (Grosse-Wilde et al., 2007).

**Figure 4:**
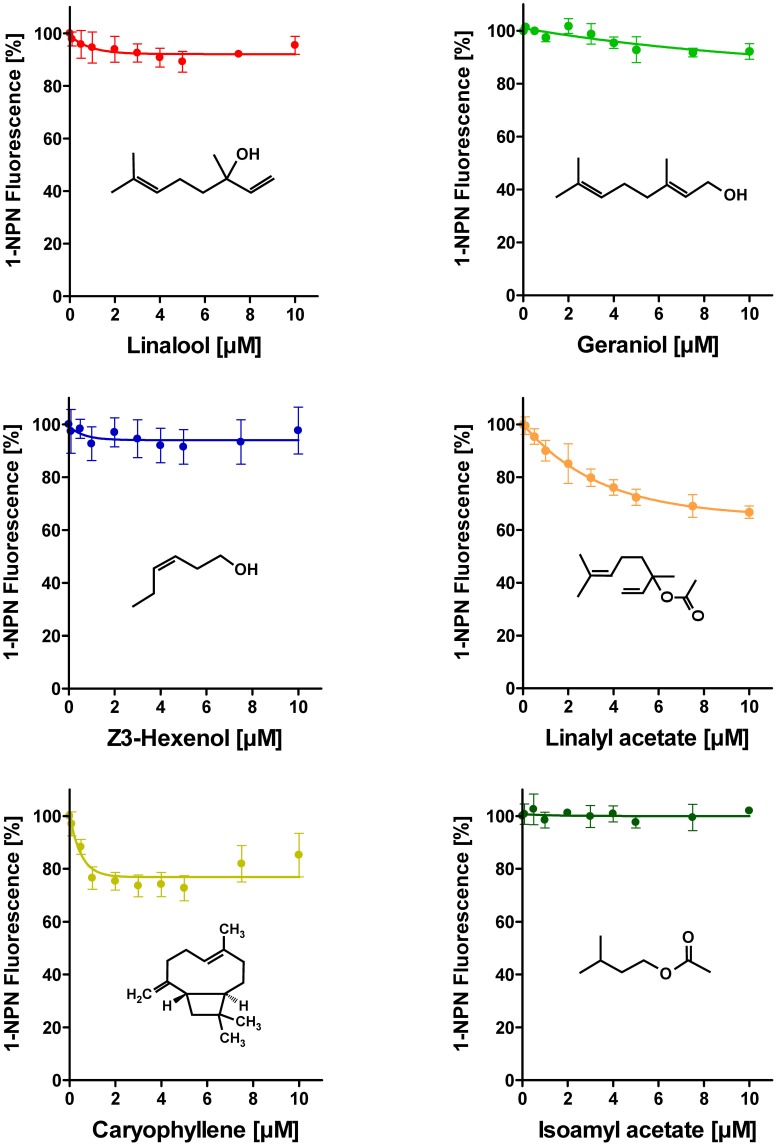
**Plant odorants do not bind or bind only very weakly to HvirPBP2.** In competitive fluorescence-binding assays, a mixture of HvirPBP2 and 1-NPN (both at 2 μM) was titrated with increasing concentrations of the plant odorants indicated, while the emission of 1-NPN fluorescence was monitored. Maximum fluorescence intensities are reported as percentages of the value in the absence of competitor (plant odorant). Data represent the mean of three independent measurements. Error bars indicate standard deviations.

To address the question if plant odorants are able to occupy the binding pocket of HvirPBP2, we tested the ability of different plant volatiles to displace 1-NPN. In most cases, plant odorants did not markedly decrease 1-NPN fluorescence even at the highest concentration (Figure 4). Displacement was seen only after application of higher doses of linalyl acetate or β-caryophyllene. Together these results indicate that plant odorants do not (linalool, geraniol, Z3-hexenol, and isoamyl acetate) or only very weakly (linalyl acetate and β-caryophyllene) bind to the hydrophobic binding pocket of HvirPBP2.

### Plant odorants do not alter Z11-16:Ald binding to HvirPBP2

**Figure 5:**
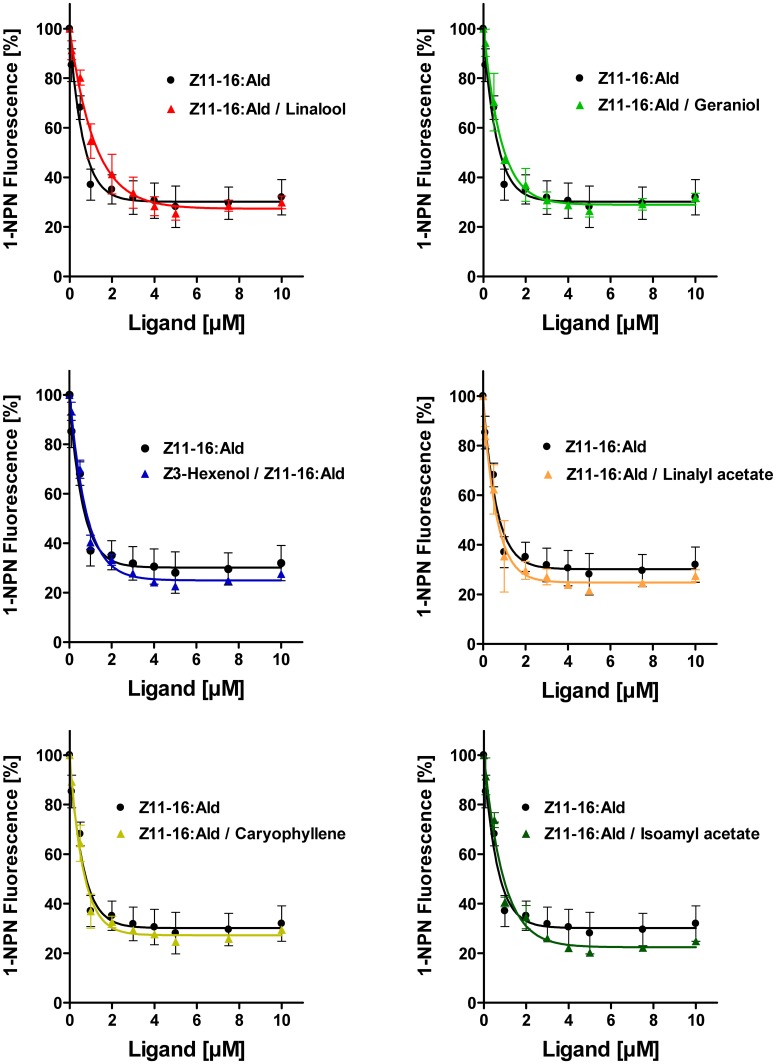
**Plant odorants do not interfere with the binding of Z11-16:Ald to HvirPBP2.** Competitive fluorescence binding assays were performed, employing HvirPBP2 and 1-NPN, both at 2 μM concentration in Ringer solution. The maximum emission of 1-NPN fluorescence was monitored after increasing concentrations (0–10 μM) of Z11-16:Ald were added and the plant odorants indicated (1:1 ratio). Maximum fluorescence intensities over concentration are shown as percentages of the value in the absence of the mixture. For comparison, the displacement curve determined for the pheromone component alone is depicted in addition to the displacement curves for the mixtures.

Despite the inability to displace 1-NPN, it is possible that plant odorants could affect the Z11-16:Ald binding of HvirPBP2 in a different way; for example, acting as allosteric effectors plant odorants may bind outside the Z11-16:Ald binding pocket and cause conformational changes of HvirPBP2, which may alter pheromone binding in a non-competitive manner. Searching for possible non-competitive effects of plant odorants on pheromone binding to HvirPBP2, we tested mixtures of Z11-16:Ald and odorants in a second series of 1-NPN displacement experiments. When the displacement curves for Z11-16:Ald alone are compared to the curves obtained for pheromone plus plant odorant, no statistically significant difference in the binding curves were found (Figure 5). From these experiments we conclude that these plant odorants do not interfere with the ability of pheromones to bind to HvirPBP2 thus, indicating that it seems not to be a perturbed pheromone-binding protein which causes a plant odorant-mediated attenuation of the pheromone-induced response of Ph-OSNs on the antenna (Hillier and Vickers, 2011) and in the cumulus region of the AL (Figures 1, 2).

### Plant odorants affect the pheromone-induced response of HR13-expressing cells

**Figure 6:**
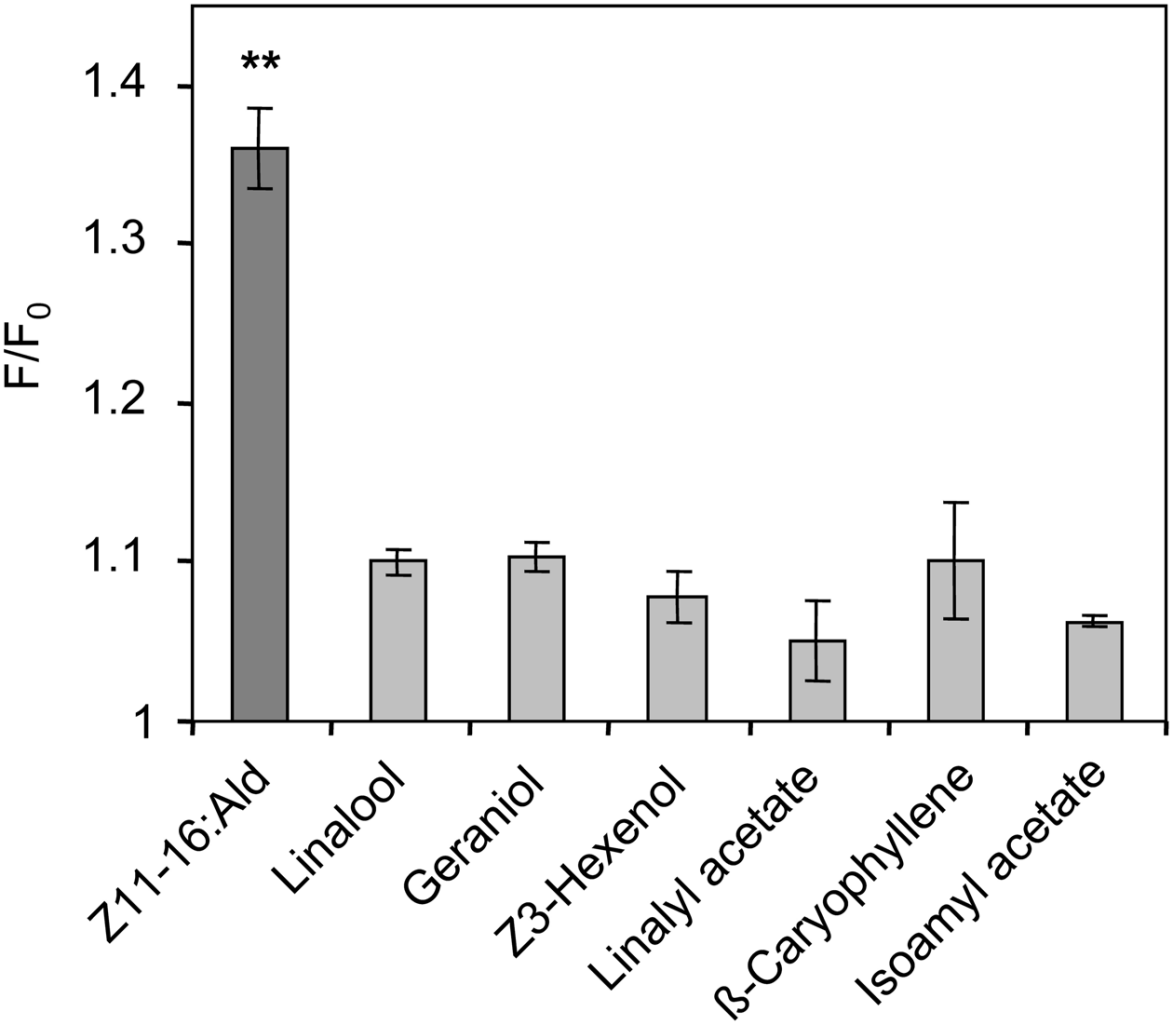
**Responses of HR13-expressing cells to Z11-16:Ald and plant odorants.** HR13 cells respond to stimulation with Z11-16:Ald (1 nM) but not to linalool, linalyl acetate, Z3-hexenol, geraniol, isoamyl acetate, and β-caryophyllene even at 100-fold higher doses (100 nM). Data represent the mean calcium responses of cells expressed as *F*/*F*_0_ ± SE ratios determined from at least three independent experiments with a minimum of 30 cells each. Data were normalized against control measurements using Ringer with 0.1% DMSO and 0.1% *n*-hexane for stimulation. Responses which differed significantly from those of the control are indicated by asterisks (^**^p < 0.01; One-Way ANOVA followed by Dunnett's post-test).

To determine whether plant odorants may affect the PR for Z11-16:Ald on the antenna, we next examined whether a HR13-mediated pheromone response is altered in the presence of plant odorants. We used HEK293/Gα15 cells stably expressing HR13 and performed fura-2-based calcium imaging experiments in order to compare the responsiveness of the cells upon stimulation with the pheromone component or pheromone/plant odorant mixtures. In a first set of experiments we monitored changes in the level of intracellular [Ca^2+^] of HR13 cells after stimulation with plant odorants used in the AL experiments (see above). Previous dose-response experiments (Grosse-Wilde et al., 2007) had shown that the threshold concentration for stimulating HR13 cells with Z11-16:Ald solubilized with DMSO in Ringer solution was about 10 pM. To detect any possible response of HR13 cells to plant odorants, we therefore used a 10,000-fold higher odorant concentration (100 nM). Stimulation of the HR13 cells with 100 nM of the various plant odorants did not elicit any calcium signals that differed significantly from the control (Figure 6). In accordance with previous work (Grosse-Wilde et al., 2007), cells stimulated with a 1 nM solution of Z11-16:Ald revealed a clear calcium response (Figure 6); such a response indicates the presence of a functional HR13 receptor protein, which binds the pheromone component and activates reaction cascades leading in turn to a rise in intracellular [Ca^2+^].

**Figure 7:**
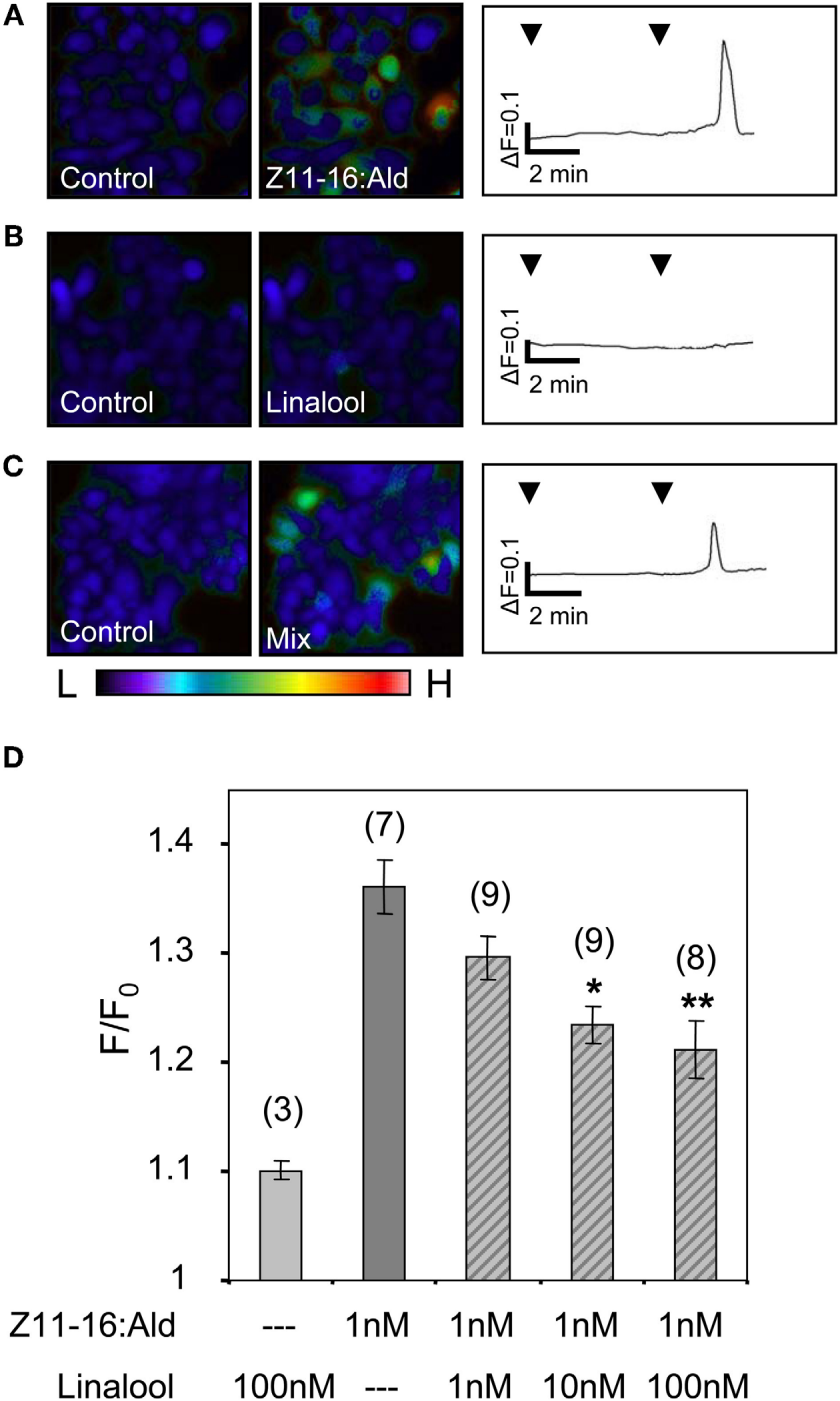
**Linalool reduces the responses of HR13-expressing cells to Z11-16:Ald. (A–C)** Pseudocolor images on the left indicate calcium levels in HR13-expressing cells after the application of Ringer with 0.1% DMSO, 0.1% *n*-hexane (control) or stimulation with solutions containing 1 nM Z11-16:Ald **(A)**, 100 nM linalool **(B)** or a mixture of both **(C)**. The color bar indicates low (L) and high (H) calcium concentration in blue and red, respectively. Calcium responses of representative cells from the experiments are shown to the right as changes of fura-2 fluorescence intensity ratios (340/380 nm) over time. HR13-expressing cells displayed clear calcium responses to Z11-16:Ald **(A)**, whereas these cells did not respond to linalool **(B)** and showed reduced responses to a mixture **(C)** of the pheromone component and the plant odorant (ratio 1:100). **(D)** Responses of HR13-expressing cells to Z11-16:Ald/linalool mixtures at different ratios. Cell responses were monitored after stimulation with solutions containing 1 nM Z11-16:Ald and 1, 10 or 100 nM linalool, respectively. (For comparison, data for linalool and pheromone component alone were adopted from Figure 6.) HR13 cells do not respond to linalool (100 nM) but show a clear calcium signal after stimulation with Z11-16:Ald (1 nM). The pheromone-induced calcium response of the cells is significantly reduced in the presence of a 10- and 100-fold excess of linalool. Bars represent the mean responses of cells reported as *F*/*F*_0_ ± SE ratios determined from 3 to 9 independent replicates with at least 30 cells in each experiment. Values have been normalized to the control. Responses to mixtures, which were significantly decreased compared to the response to the pheromone component alone, are indicated by asterisks (^*^p < 0.05, ^**^p < 0.01; One-Way ANOVA followed by Dunnett's post-test).

Next, we analyzed the responses of HR13 cells to a stimulation with mixtures of Z11-16:Ald and single plant odorants. First, the odorant linalool was tested, which caused a strongly attenuated pheromone response at the level of the AL (Figures 1, 2). Stimulating HR13-expressing cells with 1 nM Z11-16:Ald elicited a clear calcium response (Figure 7A), while linalool (100 nM) alone did not alter their calcium levels (Figure 7B). Interestingly, simultaneous application of Z11-16:Ald and linalool led to a significantly weaker calcium response (Figure 7C). To confirm the specificity of the linalool effect we used different ratios of Z11-16:Ald to plant odorant (1:1, 1:10, and 1:100) (Figure 7D). The results revealed that the pheromone-induced calcium responses of HR13 cells were significantly reduced at 10- and 100-fold excess of plant odorants. Even a 1:1 ratio of pheromone component to plant odorant resulted in a weaker, though not significant calcium signal. Thus, linalool reduced the pheromone-induced calcium response of HR13 cells in a dose-dependent manner.

**Figure 8:**
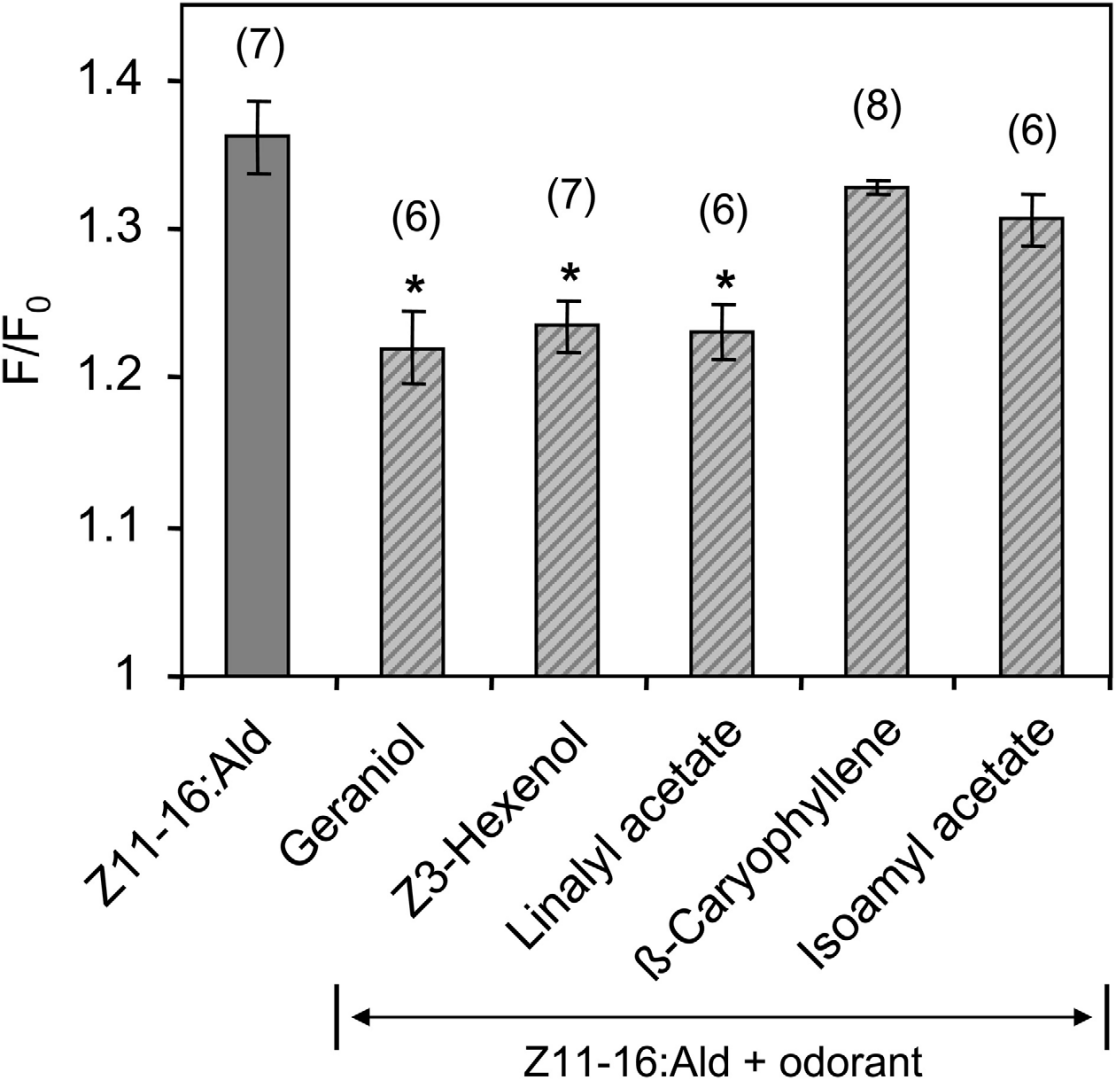
**Responses of HR13-expressing cells to mixtures of Z11-16:Ald with different plant odorants.** HR13 cells were stimulated with solutions containing 1 nM Z11-16:Ald and 100 nM of the respective plant odorant (1:100 ratio). The pheromone response was significantly reduced in the presence of linalyl acetate, Z3-hexenol, and geraniol, but not in mixtures containing isoamyl acetate and β-caryophyllene. Data represent the mean calcium responses of cells expressed as *F*/*F*_0_ ± SE ratios determined from 6 to 8 independent experiments with a minimum of 30 cells each. Data were normalized to the control. Asterisks indicate mixture responses, which differed significantly from the responses to the pheromone component alone (^*^p < 0.05; One-Way ANOVA followed by Dunnett's post-test).

In a further series of calcium imaging experiments, we tested if other plant odorants that suppressed pheromone-induced activity in the cumulus region of the MGC (Figure 2) also affected the pheromone responses of HR13 cells. We found that a mixture containing 100-fold excess of linalyl acetate, Z3-hexenol, or geraniol significantly reduced the pheromone-induced calcium signal (Figure 8). In contrast, the odorant isoamyl acetate, which did not affect pheromone-evoked signals in the MGC, did not significantly change the pheromone-induced calcium responses of HR13 cells. Similarly, β-caryophyllene did not alter the pheromone-induced response (Figure 8). Together these results suggest that in *H. virescens*, the plant odorant-provoked suppression of pheromone-induced firing of Ph-OSNs as reported by Hillier and Vickers (2011) and the inhibition of the pheromone-induced response in the Ph-OSNs projection area in the AL are mainly due to a plant odorant-dependent interference at the level of the PRs.

## Discussion

### Plant odorants suppress pheromone-evoked activity in the antennal lobe

In this study we examined the effect of plant odorants on peripheral detection and primary central coding of a sex pheromone component using the noctuid moth *H. virescens* as a model. Functional imaging studies in the moth AL revealed that stimulation of the male antenna with the major sex pheromone component, Z11-16:Ald, in the presence of distinct plant volatiles, namely linalool, linalyl acetate, Z3-hexenol, and geraniol resulted in a significantly reduced pheromone-induced calcium signal in the cumulus region of the MGC, the projection area of Z11-16:Ald-specific Ph-OSNs. In contrast, the odorant isoamyl acetate did not have a significant effect. Interestingly this odorant is the only fruit odorant in our stimulus set and might not be of ecological relevance for a male moth, while the other compounds are emitted by flowers or leafs. *In vivo* calcium imaging using bath-applied Calcium Green™ allowed us to monitor spatio-temporal changes in intracellular calcium levels in the AL, mainly reflecting the presynaptic calcium influx into OSNs (Galizia et al., 2000; Bisch-Knaden et al., 2012). In line with this observation, previous single sensillum recordings from the antenna of *H. virescens* (Hillier and Vickers, 2011) revealed that stimulation with mixtures of the pheromone component and linalool or Z3-hexenol strongly reduced the spiking activity of Z11-16:Ald-specific Ph-OSNs. Contrary to *H. virescens* these plant odorants act synergistically with Z11-16:Ald in the heliothine moth *Helicoverpa zea* leading to an increased spiking activity (Ochieng et al., 2002). Whether these differences in mixture responses in the two heliothine species may be due to differences in their odorant receptors for Z11-16:Ald or result from other mechanisms have yet to be identified. Mentionable, in *H. virescens* an increase in spike frequency of Ph-OSNs after stimulation with a mixture of Z11-16:Ald and β-caryophyllene was noted (Hillier and Vickers, 2011). We did not test this compound in our AL experiments but found no β-caryophyllene-produced synergy in our experiments with HR13-expressing cells, suggesting that the plant odorant elicits a synergistic effect via a HR13-independent mechanism.

Interestingly, and similar to the results for linalool and Z3-hexenol in *H. virescens*, a reduction of Ph-OSN spiking and a suppression of the pheromone-evoked activity in the AL was recently found for the plant odorant heptanal in the moth *Agrotis ipsilon* (Deisig et al., 2012). Although the reduced firing rate of OSNs correlate with reduced responses in the MGC we cannot rule out the possibility that inhibitory neural circuits, mediated by GABAergic local interneurons in the moth AL, also contribute to the observed inhibition of pheromone-evoked signals. Since local interneurons form multiglomerular wide-field arborizations and connect the MGC with ordinary glomeruli (Christensen et al., 1993; Anton et al., 1997; Seki and Kanzaki, 2008), they might inhibit the MGC when a plant odor is applied. However, since our data strongly suggest that the inhibitory effect is already taking place at the PR site, we assume that the contribution of the inhibitory AL network to the observed effect is probably rather minor. Nevertheless, we will silence GABA-mediated inhibition in the AL in future experiments to investigate its contribution or feedback signaling.

### Plant odorants interfere with pheromone binding to HR13

Our data indicate that the attenuating effect of plant odorants in detection of the major sex pheromone component occurs at the level of the PR HR13. This is reminiscent of recent findings of the fruit fly *Drosophila melanogaster* and the mosquitoes *Anopheles gambiae* and *Aedes aegypti*: in these insects, the responses of various olfactory receptor (OR) types to odorants were inhibited in the presence of several insect repellents (Ditzen et al., 2008; Bohbot and Dickens, 2010, 2012; Bohbot et al., 2011). For some mosquito ORs the data suggest a competitive antagonism or an allosteric inhibition of the repellents. Both mechanisms could also account for the interference of plant odorants with the Z11-16:Ald response; plant odorants could occupy the pheromone binding site of the HR13 receptor or affect the receptor activity by allosteric inhibition.

Using a competitive binding assay, we confirmed and extended previous results demonstrating that HvirPBP2 is the binding protein for the major sex pheromone component (Grosse-Wilde et al., 2007). In contrast, none of the plant odorants was bound or did affect the binding of the pheromone component to HvirPBP2. These results suggest that plant odorants do not interfere with the solubilization and transfer of the major sex pheromone component in the sensillum lymph.

The finding that HvirPBP2 does not bind non-pheromone odorants in its ligand binding pocket raises the question of how inhibitory plant odorants overcome the aqueous sensillum lymph to elicit their effects at the receptor site. Although some of the compounds used here—for example, linalool—are soluble in aqueous solutions, others are hardly soluble or even non-soluble, e.g., linalyl acetate. Conceivably, the transfer of such compounds may be mediated by other proteins present in the sensillum lymph surrounding the HR13-expressing Ph-OSN. In support of this notion, previous *in situ* hybridization studies have shown that HvirPBP1 is co-expressed with HvirPBP2 in support cells associated with the same sensillum (Grosse-Wilde et al., 2007) and three PBPs coexist in pheromone responsive hairs of *Antheraea polyphemus* (Forstner et al., 2009). In addition, certain sensilla in the silk moth *Bombyx mori* co-express BmorPBP and the antennal binding protein X (ABPX) (Maida et al., 2005). Thus, HvirPBP1 or other yet not identified PBPs and OBPs coexisting in the sensillum lymph with HvirPBP2 may account for the solubilization and transfer of plant odorants.

Although non-pheromone odorants do not bind to the pheromone-binding pocket it cannot be excluded that they may interact with the surface of HvirPBP2 and thus be transported through the sensillum lymph. Considering such a possibility, one has to take into consideration that a plant odorant/PBP interaction may block conformational changes, which may be necessary for pheromone release (Wojtasek and Leal, 1999) or receptor activation by PBP/ligand complexes (Laughlin et al., 2008). In this way, the plant odorant/PBP interaction could directly contribute to the suppression of pheromone-evoked responses observed in single sensillum recordings (Party et al., 2009; Hillier and Vickers, 2011; Deisig et al., 2012) and calcium imaging of the AL (this study).

### Ecological relevance of pheromone/plant odorant interference

Female-released pheromones trigger and control upwind flight behavior and guide the male to the mating partner. According to our study and the work of others, the sex pheromone detection system of male moths seems unexpectedly susceptible to plant odorants in the environment. Most studies have reported that pheromone detection is suppressed in the presence of plant odorants (Party et al., 2009; Hillier and Vickers, 2011; Deisig et al., 2012).

With regard to a sensitive detection of the female-released pheromone and mate localization, the mostly found inhibition of the male pheromone detection system by plant odorants appears to be counterproductive. However, data suggest that suppression of the pheromone response by plant odorants may be of advantage. In electrophysiological studies of male antennae a background of plant odorants decreased the intensity of pheromone signals and improved the separation of pheromone pulses by the Ph-OSNs (Party et al., 2009). Furthermore, due to the reduced response rate both during and between pheromone pulses, a plant odorant background contributes to preserve the temporal structure of the pheromone signal (Rouyar et al., 2011). Because information encoded in the temporal structure of a pheromone plume is particularly important for orientation of male moths toward a pheromone source (Vickers, 2006), a higher odor background in the vicinity of a calling female sitting on a plant may positively affect mate localization by males approaching her.

### Conflict of interest statement

The authors declare that the research was conducted in the absence of any commercial or financial relationships that could be construed as a potential conflict of interest.

## Abbreviations

AL: antennal lobe
HR: *Heliothis virescens* receptor
PBP: pheromone binding protein
Z11-16:Ald: (Z)-11-hexadecenal
OSNs: olfactory sensory neurons
Ph-OSNs: pheromone-responsive olfactory sensory neurons.
